# A Rare Case of Acute Pancreatitis as Dengue Complication

**DOI:** 10.1155/2023/2619785

**Published:** 2023-03-06

**Authors:** Tuy Hong Thi Nguyen, Hien Quang Nguyen

**Affiliations:** ^1^Tam Anh Hospital, Ho Chi Minh City, Vietnam; ^2^University of Medicine and Pharmacy, Ho Chi Minh City, Vietnam

## Abstract

A 31-year-old male was admitted to the hospital because of fever for 2 days. He also had chills, headaches, muscle aches, fatigue, and diarrhea. His vital signs were stable. Dengue virus nonstructural protein 1 (NS1) antigen was positive. Laboratory tests were significant for thrombocytopenia of 67.000/mm^3^ and high hematocrit of 45%. On the fifth day of the onset of fever, he experienced sudden epigastric pain. Laboratory results showed elevated serum amylase and lipase. Noncontrast abdominal CT findings were consistent with acute pancreatitis, Balthazar grade D. The patient was managed with supportive care and bowel rest. Two days later, his condition became stable, and he was discharged without complications.

## 1. Introduction

Dengue is a viral disease, transmitted by *Aedes* species mosquito, endemic to parts of Asia and South America, among which Vietnam is one of the countries carrying the highest burden, reaching more than 100,000 caseson average every year. The highest incident was 320,331 casesin 2019, with 53 fatalities. There are four serotypes of the virus belonging to the *Flaviviridae* family that cause dengue. The majority of dengue virus infection cases are asymptomatic or present with mild symptoms. However, dengue also can manifest as a severe illness, including hemorrhage or shock syndrome, and in rare cases, it is fatal [1–3]. Although the diagnosis of dengue is not difficult, especially in the endemic areas, and in the season when the disease surges, early recognition of severe conditions is paramount in treatment and prognosis. We report a severe dengue case, complicated with acute pancreatitis, a very rare and atypical clinical association.

## 2. Case Presentation

A 31-year-old male patient with no prior medical history presented to the emergency room with high-grade fever for 2 days. He also had chills, headaches, muscle aches, fatigue, and diarrhea. He used over-the-counter acetaminophen to control his symptoms.

On admission, the patient was oriented. His temperature was 38°C, blood pressure was 130/90 mmHg, pulse was 92 bpm, respirations were 20 bpm, and oxygen saturation was 98%. Physical examination showed no abdominal pain, no organomegaly, a negative Murphy's sign, and no evidence of mucocutaneous bleeding. Laboratory tests were significant for thrombocytopenia with platelet count of 67.000/mm^3^, high hematocrit (45%), hemoglobin level of 15.2 g/dL, leukocyte count of 3.310/mm^3^, and a positive NS1 antigen test. His liver enzymes were mildly elevated, AST was 89 U/L, and ALT was 69 U/L; serum HbsAg and HCV antibody were negative. Serum creatinine and C-reactive protein were within normal limits. Abdominal ultrasound showed normal liver and pancreas sizes, thickening gallbladder wall (8 mm), and no intraperitoneal fluid was noted. Chest X-ray was unremarkable.

**Figure 1:**
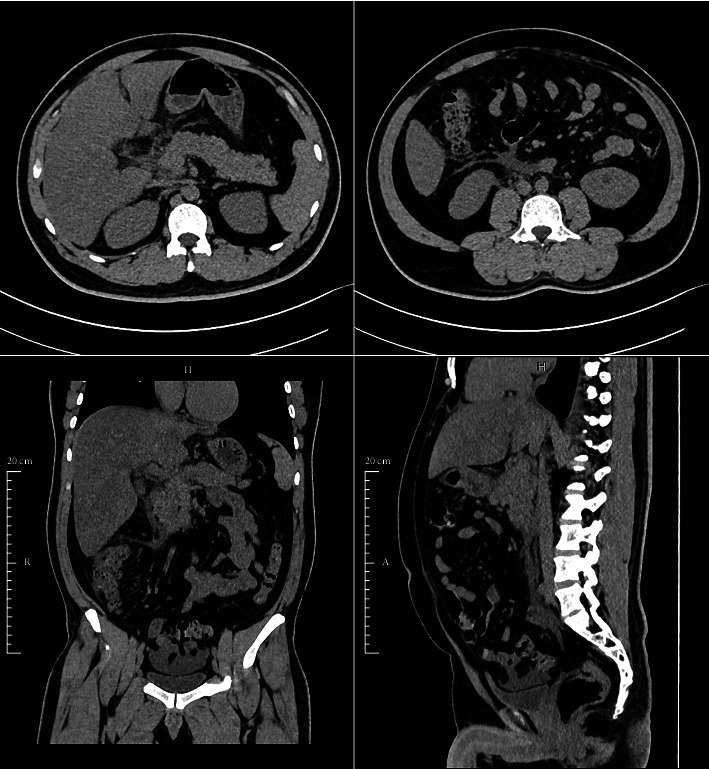
Noncontrast abdominal CT showing an enlarged pancreas, peripancreatic fat stranding, and peripancreatic fluid collection, consistent with Balthazar score D.

In the hospital, his diarrhea was worsening, more than 5 times a day. On the fifth day after the onset of fever, he started to have burning-like abdominal pain, which was localized in the epigastrium and radiated to the back. He had 2-3 episodes of nausea and vomiting per day. He also developed petechiae over his lower limbs. Laboratory results showed elevated serum amylase (174 U/L) and lipase (606 U/L). Platelet count decreased to 10.000/U, hematocrit was 51%, leukocyte count was 5.200/mm^3^, and serum triglyceride was 3.81 mmol/L (Table 1). Plain abdominal computed tomography revealed an enlarged pancreas, peripancreatic fat stranding, and peripancreatic fluid collection (Balthazar score D) along with a minimal right pleural effusion (Figure 1). He was managed with intravenous fluids, analgesics, antacid, and bowel rest. Two days later, his clinical condition became stable, he started to be afebrile, the abdominal pain was resolved, and his platelet cell count was trending up. Subsequently, he was discharged without complications.

## 3. Discussion

This case report emphasizes the notability of the diagnosis of acute pancreatitis in patients with dengue who abruptly develop severe abdominal pain. Our patient was admitted to the hospital with fever and severe thrombocytopenia. A positive NS1 test result confirmed dengue. During hospitalization, he developed sudden onset of abdominal pain along with elevated amylase and abnormal abdominal CT, which indicated the presence of pancreatitis. This association is unusual, with only a few cases were represented in the literature [2].

The two most common etiologies associated with acute pancreatitis are gallstones and alcohol consumption. Cholelithiasis is seen in approximately 40–70% of cases, while alcoholism is present in 25–35% of cases. Less frequent causes include trauma, medications, autoimmune diseases, congenital abnormalities, hypercalcemia, or infections [4, 5]. There are many organisms associated with infectious pancreatitis, including parasites, bacteria, and viruses. A notable pathogen in the tropical areas is *Ascaris lumbricoides*, which induces pancreatitis by obstruction of the biliary and pancreatic duct. In particular, viruses are the most common microbes that account for infectious pancreatitis, including hepatitis viruses, Coxsackie virus, mumps, varicella-zoster virus, CMV, EBV, HSV, and HIV [6].

The mechanism of viral-induced pancreatitis is still controversial. Many researchers have provided different hypotheses. Pancreatic islet cells can be destroyed directly by viruses, as well as by inflammatory reactions, or cellular edema in response to viral infection. This hypothesis is supported by autopsies showing that HBV antigen present in the cytoplasm of exocrine pancreatic cells. Pancreatic enzymes from injured cells can be leaked and can provoke pancreatic necrosis. Another explanation is that the ampulla of Vater or the pancreatic duct has gotten edema, leading to the obstruction of the outflow of pancreatic fluid [7, 8]. It is reasonable that multiple mechanisms can contribute to pancreatitis at the same time, as we believe in our case of dengue. However, it can be challenging to definitively determine whether pancreatitis is a complication of dengue or merely a coincidence. The association between the two is rarely reported to establish a statistically significant correlation. Nonetheless, as we mentioned, there is a reasonable hypothesis about how viruses can induce pancreatitis, and therefore, a causative relationship between the two clinical presentations is believed to exist.

Imaging has indispensable values in the assessment of organ involvement in dengue. On ultrasonography, gallbladder wall thickening (GBWT) can be a useful screening finding for dengue as well as a predictive factor for severity and prognosis. GBWT is an indicator of increased capillary permeability, the pathophysiology of dengue. A GBWT of more than 3 mm has a high sensitivity for detecting dengue. GBWT greater than 3 mm is associated with more severe cases, and patients with GBWT above 5 mm increase risk of developing hypovolemic shock [9, 10]. Our patient with GBWT of 8 mm had a higher risk of severe complications. Performing ultrasonography of the pancreas in patients with dengue hemorrhagic fever, Setiawan reported an enlarged pancreas was found in 29% of cases, 14% of whom had mild and 44% of whom had severe clinical presentations. The pancreas was hyperechoic compared with the liver in 25% of cases, isoechoic in 69%, and hypoechoic in 6% of cases [11]. Therefore, when a patient with dengue presents with abdominal pain, it is important to perform imaging studies such as ultrasonography and CT scans, if available, to detect early organ involvement and enable effective management. It is noteworthy that in dengue-endemic countries of South and Southeast Asia, many clinical centers may not have access to CT scans. In these situations, diagnosis may rely on clinical signs, including typical abdominal pain and elevated pancreatic enzymes.

Treatment of dengue-induced pancreatitis is similar to that in other etiologies, with primary management being supportive and bowel rest. Our patient had mild pancreatitis and recovered 2 days after the onset of epigastrium pain. To date, no predisposing factors have been identified to predict the onset of pancreatitis in patients with dengue. This complication may occur unpredictably, emphasizing the importance of early detection and providing adequate supportive treatment, which plays the most crucial and effective role.

## 4. Conclusions

Acute pancreatitis should be considered in patients with dengue, who presents with sudden new onset of severe abdominal pain. Pancreatic imaging and amylase levels are the next steps in management. Appropriate early intervention plays a vital role in treatment and recovery.

## Figures and Tables

**Table 1:** Clinical and laboratory results on admission and 3 days after hospitalization.

	On admission	3 days after hospitalization
Clinical signs	(i) Fever (day 2)	(i) Fever (day 5)
	(ii) Diarrhea	(ii) Diarrhea worsening
	(iii) Chills, headaches, muscle aches, and fatigue	(iii) Chills, headaches, muscle aches, and fatigue continuing
		(iv) Sudden epigastric pain, nausea, and vomiting

Plt (k/*μ*L)	67	10

Hct (%)	45	51

HGB (g/dL)	15.2	17.4

WBC (k/*μ*L)	3.31	5.2

Others	(i) NS1 : positive	(i) Serum amylase: 174 U/L
	(ii) AST: 89.1 U/L	(ii) Serum lipase: 606.6 U/L
	(iii) ALT: 69.7 U/L	

## Data Availability

The data used to support the findings of this study are included within the article.
